# Association of Mammographic Breast Density with Dairy Product Consumption, Sun Exposure, and Daily Activity

**DOI:** 10.1155/2014/159049

**Published:** 2014-03-04

**Authors:** Sadaf Alipour, Azin Saberi, Afsaneh Alikhassi, Leila Bayani, Ladan Hosseini

**Affiliations:** ^1^Surgery Department, Arash Women's Hospital, Tehran University of Medical Sciences, Tehran, Iran; ^2^Vali-e-Asr Reproductive Health Research Center, Tehran University of Medical Sciences, Tehran, Iran; ^3^Radiology Department, Cancer Institute, Tehran University of Medical Sciences, Tehran, Iran; ^4^Radiology Department, Arash Women's Hospital, Tehran University of Medical Sciences, Tehran, Iran; ^5^Research Development Center, Arash Women's Hospital, Tehran University of Medical Sciences, Tehran, Iran

## Abstract

*Background*. Mammographic density is a risk factor, for breast cancer and its association with various factors is under investigation; we carried out a study to assess its relationship with daily dairy intake, sun exposure, and physical activities. *Patients and Methods*. Women ≥40 years of age were interviewed about habits of dairy product consumption, daily sun exposure and physical activity. Exclusion criteria consisted of history of breast cancer, consumption of calcium and vitamin D supplements, hormone replacement therapy, or renal disease. Mammographic densities were classified according to the classification system of the American College of Radiologists into 4 classes. *Results*. Overall 703 cases were entered in the study. The mean age was 48.2 ± 6.2 years. The most common and least frequent classes of mammographic density were classes 2 and 4, respectively. There was no significant association between mammographic density and rate of dairy consumption, amount of sunlight exposure, and daily physical activity. *Conclusion*. Relation of sunlight exposure and intake of milk products with mammographic density need further study, while the subject of physical activity can be evaluated by a systematic review and meta-analysis of the existing literature.

## 1. Introduction

Density of breast parenchyma in mammography is presently a feature recognized as one of the risk factors for breast cancer [1–3]. Relative risk for breast cancer in high density mammograms is 2- to 6-fold higher than in low density ones [1, 2, 4].

Among factors recognized as potentially capable of influencing mammographic density (MD), hormonal related issues [5, 6], body mass index [7, 8], age [9], and some dietary factors have been implicated [10–12]. As well, other likely related topics, including anthropometric characteristics [13], smoking [14, 15], metabolic syndromes [16], sun exposure [17], and physical activity [18, 19], have been investigated.

We conducted a study to determine the association of mammographic breast density with habits of dairy intake, daily sun exposure, and rate of daily activities.

## 2. Methods

This is a cross-sectional study completed as component of a large study being carried out in the Breast Clinic of Arash Women's Hospital, Tehran, Iran. Women aged 40 years or above who were candidates for breast cancer screening were entered in the study and those who were practicing sports or had a personal history of breast cancer, recent consumption of calcium supplements, vitamin D, or hormone replacement therapy (HRT), or any kind of renal disease were excluded. A questionnaire about habitual daily sun exposure, patterns of consumption of dairy products, and amount of daily activity of participants was filled out by a trained interviewer. Following clinical breast examination, all of them underwent screening mammography by standard bilateral mediolateral-oblique and craniocaudal views. Two expert radiologists classified the density of mammograms according to the Breast Imaging-Reporting and Data System (BIRAD) of parenchymal mammographic classification system of the American College of Radiologists (ACR) into *class 1*: almost entirely fatty (<25% glandular), *class 2*: scattered fibroglandular densities (approximately 25–50% glandular), *class 3:* heterogeneously dense (approximately 51–75% glandular), and *class 4*: extremely dense [8, 20].

Daily dairy product consumption was identified as number of servings per day. Due to the lack of a defined food-composition table for Iran [21], equivalent amounts of milk products making one serving were described in agreement with Canada and the United States' food guide [22, 23] as well as habitual Iranian dietary patterns of eating. Each serving was assumed equal to one glass (250 cc) of milk, 40–50 grams of cheese (the size of one box of matches in Iranian cheese products), one cup (175–200 cc) of yogurt, one glass of doogh (Iranian yogurt drink composed of yogurt and water with the same consistency as milk in terms of constituents and proportions), or one glass of ice cream [24, 25]. Rate of daily consumption was classified as less than one, 1 to 3, or more than 3 servings per day.

The lifestyle of most urban Iranian women is such that they stay indoors through a great proportion of the day. Therefore, we classified the time of sun exposure to less or more than half an hour in daylight each day.

Women in Iran are rather active in house work, and the small proportion which are employed or have a business outdoors accomplish their household duties to the same extent as housewives. Accordingly, daily activity was classified as mild, moderate, or severe as follows. Mild activity constituted of only house work in a small or moderate-size house or house work with help and support from others in a large house. Moderate activity was defined as house work with the same description of the mild group in addition to outdoors work or nonsupported house work in a large house. Severe activity was depicted as any amount of work more than the above.

Association between MD and the three investigated parameters was analyzed by SPSS version 16. For statistical significance, *χ* ^2^ test was used and *P* < 0.05 was regarded as statistically significant. The study was approved by the Ethics Institutional Review Board of Tehran University of Medical Sciences.

## 3. Results 

After excluding 135 of the participants according to the exclusion criteria, 703 cases were entered in the study. The mean age was 48.2 ± 6.2 years.  Table 1 demonstrates the frequency of different classes of MD in each category of investigated variables.

Among all classes of MD, the most frequent in all participants and in each studied group was class 2 and the second most common was class 3. As well, in all groups, the least frequently detected MD was class 4, except for those who consumed more than 3 servings per day of milk products; class 1 MD was the least frequent in this group. Moreover, the only participants with a high gap between class 4 and 1 were women with mild daily physical activity.

Analysis showed no significant association between MD and milk product intake whether the participants consumed less than one, one to 3, or more than 3 servings of the products per day (*P* = 0.632). Likewise, the distribution of classes of MD was comparable in low and high amounts of exposure to sunlight and the difference was nonsignificant in the two groups (*P* = 0.283). Mild, moderate, or high levels of daily physical activities also did not have any statistically significant effect on MD (*P* = 0.304).

## 4. Discussion

The high likelihood of getting involved with breast cancer in women with dense mammograms [26] has motivated the completion of numerous researches regarding factors contributing to higher densities in the breast image. Association of MD and eating patterns has been the subject of several researches. In 1999, Knight et al. investigated the effects of ingested fat on the risk of breast cancer by assessing changes of MD after 2 years of consuming low-fat, high-carbohydrate foods. They detected a significant amount of reduction in MD after the regimen; this effect was magnified in those going through menopause in that period of time [27]. Qureshi et al. roughly confirmed these results in their study but showed no relationship between MD and ingestion of foods containing vitamins and calcium or levels of protein and carbohydrate intake [28]. Nevertheless this was not validated by Sala et al. who had previously observed increased MD in women eating more protein and carbohydrate and no association of MD with fat and vitamin intake [29]. Soy-containing foods have also been recognized as probably influencing MD [12, 30].

Among studies investigating the subject of food and MD, few have considered the relationship between MD and the rate of milk and milk products consumption; so far the issue is left unsolved although the bulk of existing literature might be mildly in favor of an inverse association. Some of the relevant studies are summarized in Table 2. The highest proportion of our study population either used low or moderate amounts of dairy products and only about 10% consumed an acceptable or high level of this group of nutrients. Nevertheless, nearly half of the latter group had class 2 MD, while less than one-third had class 3 and only about 10% proved to have very dense breasts on mammogram. These figures approximate those of the 2 other groups, establishing the lack of association between the two variables.

The relation of vitamin D levels and MD has been considered in several works; nevertheless the effect of sunlight exposure has rarely been investigated because of the complexity of its quantitative and qualitative estimation, and the results have been controversial (Table 2). In our study, MD did not differ significantly between the low- and high-sun-exposed groups. However, although Tehran is located at 35° latitude, has a temperate climate [44], and benefits from good sunlight, the majority of female residents in Tehran live indoors for the best part of the day. Consequently, even though our classification consisted of a minimum of 30 minutes per day as the threshold for high sun-exposure, less than one quartile of the study population were categorized in this group. The lack of association of MD with this parameter may be due to the low rate of exposure even in the latter group.

Physical activity is defined as any energy spending movement of the bodyand is composed of different everyday activities as recreational, occupational, or household activity [45]. This term is not the equivalent of exercise, described as a structured and rhythmic activity performed with the purpose of achieving physical fitness. The link connecting physical activity with MD has been investigated in some studies as demonstrated in Table 2, and the majority could not come across any significant association. Although the quantity of physical activity was largely different among the 3 groups in our study, it did not influence MD, confirming previous studies.

## 5. Conclusion

Our study shows no association between mammographic density and rate of dairy consumption, amount of sunlight exposure, and daily physical activity. We believe that the last issue deserves to be investigated by systematic review and meta-analysis of the existing data in the literature. Sunlight exposure and intake of calcium-containing food in regards to MD needs to be further studied.

## Figures and Tables

**Table 1:** Frequency of classes of mammographic density in each group of dairy product intake, sunlight exposure, and daily activities. The bold fonts are figures that have been specifically pointed in Section 3.

Mammographic density*	Class 1		Class 2		Class 3		Class 4		Sum	
	No	Percent	No	Percent	No	Percent	No	Percent	No	Percent within group
Dairy consumption										
Servings per day										
<1	35	12.5%	125	44.5%	100	35.6%	21	7.5%	281	42.2%
1–3	35	11.0%	163	51.3%	100	31.4%	20	6.3%	318	47.7%
>3	6	**9.0**%	33	49.3%	21	31.3%	7	**10.4**%	67	10.1%
Sunlight exposure										
Minutes/day										
<30	56	11.6%	241	49.8%	157	32.4%	30	6.2%	484	76.5%
≥30	17	11.4%	62	41.6%	57	38.3%	13	8.7%	149	23.5%
Daily activities										
Mild	9	**12.2**%	42	56.8%	22	29.7%	1	**1.4**%	74	10.7%
Moderate	51	11.3%	220	48.7%	146	32.3%	35	7.7%	452	65.2%
Severe	23	13.8%	72	43.1%	60	35.9%	12	7.2%	167	24.1%

*Class of MD according to the parenchymal mammographic classification system of ACR.

**Table 2:** Studies about association of MD with dairy product intake, sunlight exposure, or physical activity.

Authors	Studied factor	Pub yr*	Study design and details	Type of association
Bérubé et al. [31]	Calcium containing foods**	2004	(i) Semiquantitative food frequency questionnaire (ii) MD assessed by one expert reviewer evaluating percent density (iii) 1092 women 40 to 60 years old with recent 2 years screening mammogram	Inverse

Bérubé et al. [32]	Calcium containing foods**	2005	(i) 777 premenopausal and 783 post-menopausal women (ii) Food frequency questionnaire for estimation of dietary intake (iii) MD assessed by computer-assisted method	Inverse

Masala et al. [33]	Cheese ad dietary calcium	2006	(i) 2,000 women with mammogram taken 5 years after enrollment in the EPIC-Florence study (ii) Original mammograms retrieved (iii) Information on dietary habits collected during 5 years (iv) MD assessed according to Wolfe's classification	Inverse

Takata et al. [34]	Dairy products	2007	(i) 3512 mammograms from 1250 premenopausal and postmenopausal women (ii) MD assessed by computer-assisted method (iii) Validated food frequency questionnaire for estimation of dietary intake	None

Thomson et al. [10]	Dairy products	2007	(i) Cross-sectional design (ii) MD assessed by computer-assisted method (iii) Arizona food frequency questionnaire for estimation of dietary intake	Inverse

Mishra et al. [35]	Dietary calcium from childhood	2008	(i) cohort of 1161 women followed up since their birth (ii) documentation of diet at age 4 by 24 h recalls and at age 36, 43 and 53 by 5-day food records (iii) MD assessed by computer-assisted method	None***

Wu et al.**** [17]	Sunlight	2013	(i) 650 premenopausal women (ii) validated sunlight exposure questionnaire via telephone (iii) MD classified according to Tabar's classification	Inverse

Peplonska et al. [36]	Sunlight	2012	(i) cross-sectional study (ii) Investigating whether rotating night shift working in nurses was associated with MD (iii) 640 nurses and midwives 40 to 60 years (iv) MD assessed by computer-assisted method	None

López et al. [19]	Physical inactivity	2003	(i) information collected from the Chicago Breast Health Project (ii) evaluation of association of hours of daily physical inactivity (iii) MD assessed quantitatively (iv) 294 Hispanic women	None*****

Suijkerbuijk et al. [37]	Physical inactivity	2006	(i) cross-sectional study (ii) 620 women 49 to 68 years, participants of the Dutch Prospect-European Prospective Investigation into Cancer and Nutrition cohort (iii) self-administered questionnaire about duration and intensity of physical activity (iv) MD assessed by computer-assisted system	None

Siozon et al. [38]	Recreational physical activity	2006	(i) 375 white and African American women (ii) Structured questionnaire obtaining data from 5 time periods of activity: menarche to 1st screening mammography, first 3 and 10 years after menarche, the most recent 10 years and the 3 years prior to mammogram screening	None

Samimi et al. [39]	Recent physical activity	2008	(i) cross-sectional study (ii) 1,398 women in the Nurses' Health Study (iii) three groupings: premenopausal, postmenopausal without HRT, postmenopausal currently under HRT (iv) MD assessed by computer-assisted method (v) prospectively collected questionnaires	None

Oestreicher et al. [40]	Physical activity	2008	Cohort of pre- and early perimenopausal women of 4 different ethnicities	Non-significant inverse

Woolcott et al. [41]	Aerobic exercise	2010	(i) inside the ALPHA trial: randomized controlled trial (ii) 320 postmenopausal, sedentary women 50 to 74 years (iii) randomized to 45 minutes, 5 days per week aerobic exercise or control with usual life-style for 1 year (iv) MD assessed by computer-assisted system	None

Conroy et al. [18]	Physical activity	2010	(i) using longitudinal data from 1996 to 2004 (ii) multiethnic cohort of 722 pre- and early perimenopausal women participating in the Study of Women's Health Across the Nation	None

Marmara et al. [42]	Last five years recreational physical activity	2011	(i) cross-sectional study (ii) 724 women 45–67 years (iii) interview-administered questionnaire (iv) MD classified according to BIRAD classification	Inverse

Qureshi et al. [43]	Physical activity	2012	(i) women data from the Norwegian Breast Cancer Screening Program (ii) MD assessed by computer-assisted system (iii) 2218 postmenopausal women 50–69 (iv) 13-page questionnaire	None******

*Pub yr: Publication year, **primarily dairy products, ***except for a cross-sectional inverse association at age 53 years in post-menopausal women, ****They showed that sunlight exposure in childhood is more effective, *****MD slightly higher when 3.5 hr physical inactivity per day, ******probable inverse association in overweight women.
